# Heart Transplantation: A Review of Recent Advancements in Pre-, Peri, and Post-Transplant Screening and Management

**DOI:** 10.3390/biomedicines14102384

**Published:** 2026-10-19

**Authors:** Marc Ringor, Darren Nguyen, Fadel Boumelhem, Yousef Joseph, Divyansh Sharma, Jasmine Dugal

**Affiliations:** Department of Internal Medicine, University of Nevada, Las Vegas, NV 89102, USA

**Keywords:** end-stage heart failure, heart transplantation, mechanical circulatory support, left ventricular assist device, echocardiography, immunosuppressive therapy, induction therapy, maintenance therapy, cardiac allograft rejection, surveillance

## Abstract

Heart transplantation (HT) remains a critical treatment for patients with end-stage heart failure who remain unresponsive to optimized guideline-directed medical therapy. This review examines recent advancements in pre-, peri-, and post-transplant screening and management aimed at improving patient selection, transplant accessibility, and clinical outcomes. Pre-transplant strategies include comprehensive HT candidate evaluation, risk stratification, donor selection, allocation policies that priotize critically ill patients, and mechanical circulatory support (MCS) to stabilize patients awaiting transplantation. Peri-transplant advances focus on strategies to maintain hemodynamic stability, minimize ischemic injury, and maintain graft viability immediately after HT. Post-transplant management emphasizes individualized immunosuppressive regimens, cardiac allograft rejection surveillance, and the prevention and management of complications to improve graft survival and long-term patient outcomes. Despite substantial progress, significant challenges remain, particularly the shortage of suitable donor organs and the risk of immune-mediated graft rejection. Emerging approaches, including artificial intelligence, xenotransplantation, and regenerative medicine, offer promising opportunities to expand the donor pool, enhance clinical decision-making, and improve HT outcomes. This review examines the research that influences the current policies and practices involved in HT, featuring the pivotal findings as well as any shortcomings present among these studies. Ultimately, continued research and multidisciplinary collaboration in each phase of HT are essential to improve the short and long-term health outcomes of transplant candidates. By integrating established evidence-based practices with emerging technologies, clinicians may further improve equitable access, survival, and quality-of-life for patients with end-stage heart failure.

## 1. Introduction

For those who suffer from end-stage heart failure, despite optimized medical therapy, heart transplantation (HT) remains an evolving treatment option that can improve survival and functional independence. Because of this, multifaceted efforts have been made to expand its availability for the estimated 100,000 to 200,000 people a year in the United States who qualify for it [1]. Guidelines and techniques for HT have changed to widen access for those who are awaiting the procedure and to improve health outcomes for those who receive it. These include updates to the United Network of Organ Sharing (UNOS) for HT candidate selection [2,3] and the evidence-based use of mechanical circulatory support prior to transplantation, as well as post-operative multimodal immunosuppressive regimens. These innovations have led to improved mortality among those with end-stage heart failure, with 53% of recipients living 10 years or more post-transplant [4]. Despite these improvements, the shortfall of available donor allografts and post-operative complications such as immune cell- and/or antibody-mediated damage, also known as cardiac allograft rejection (CAR), remain persistent challenges [5]. To address these challenges, additional studies have attempted to further expand the pool of eligible donors, alongside medical advancements in mechanical circulatory support devices to extend survivability for those awaiting HT, as well as immunosuppressive and statin regimen optimization to reduce CAR rates. The aim of this study is to review the significant breakthroughs that established current practices before, during, and after HT to improve patient outcomes, as well as to emphasize future directions to continue advancing the field, including artificial intelligence, xenotransplantation and regenerative medicine to improve candidate survivability.

## 2. Pre-Transplant Stage

### 2.1. Candidate Evaluation, Risk Stratification and Selection for Heart Transplantation

**Figure 1:**
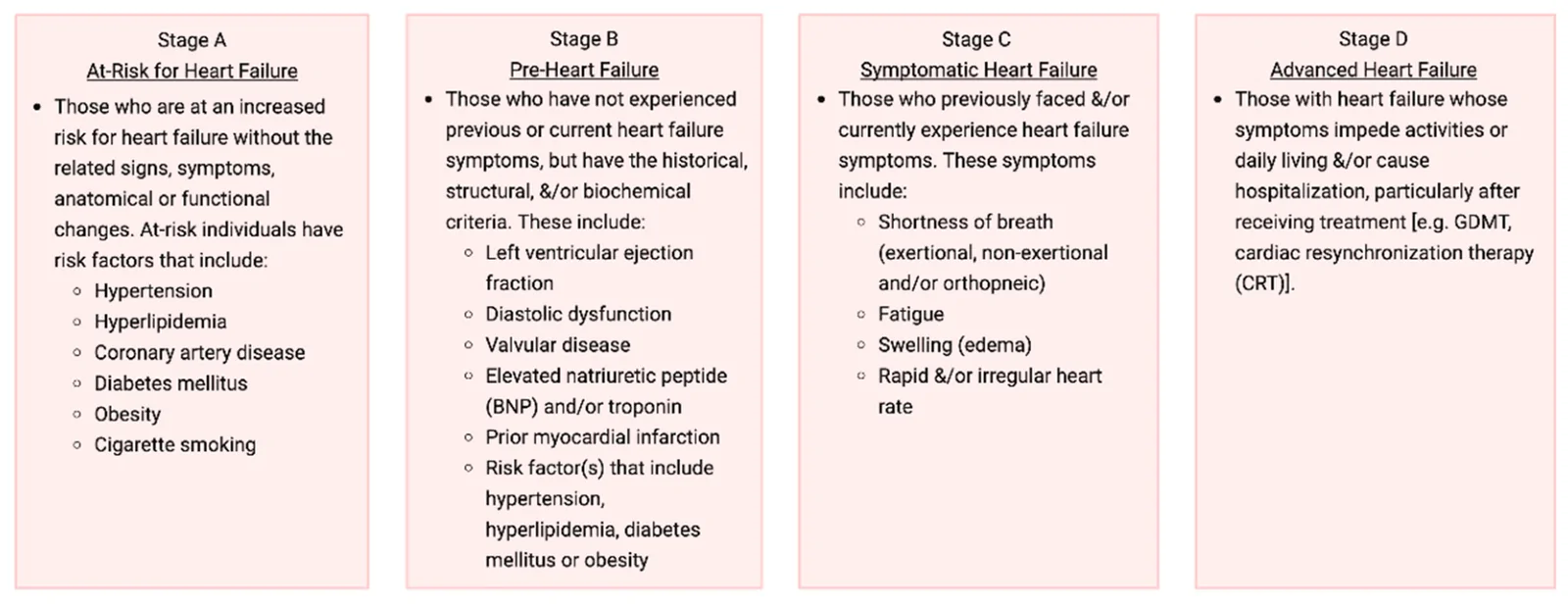
ACC/AHA/HFSA heart failure stages. Describes the four stages of heart failure (stages A to D) based on escalating criteria, including risk factors, objective findings, and classic symptoms, with those in advanced-stage heart failure remaining symptomatic despite medical intervention(s). Created in BioRender. Ringor, M. (2026). https://BioRender.com/a5kgod6 (accessed on 19 March 2026).

To ensure appropriate distribution of the limited cardiac allografts available, with priority toward those who are critically ill, major efforts have focused on enhancing the screening of potential HT candidates. For prospective recipients in the United States, the traditional first step is to stage the severity of their disease according to the American College of Cardiology, American Heart Association, and Heart Failure Society of America (ACC/AHA/HFSA) [Figure 1] [5,6]. These include stages A (at risk for heart failure), B (pre-HF), C (symptomatic heart failure), and D (advanced heart failure), with each stage stratified by risk factors, anatomical as well as biochemical changes, and increasing severity of HF symptoms, culminating in stage D, exemplified by frequent and recurrent hospitalizations despite optimized guideline-directed medical therapy (GDMT). Alongside the staging of a potential HT candidate’s heart failure is their classification according to the New York Heart Association (NYHA). These classes are based on the tolerance of heart failure patients to physical activity, from class I (able to perform activities of daily living without difficulty) to class IV (heart failure symptoms even at rest), with each class ranked by worsening independent functionality. According to the 2022 ACC/AHA/HFSA Guidelines for the Management of Heart Failure, it is a class I recommendation to affirm the goals of care and assess heart failure patients for HT after they are confirmed to have end-stage heart failure (ESHF), defined as stage D and NYHA class III with recent exacerbation (also known as IIIb) or IV heart failure [6].

Additional criteria to guide the appropriate distribution and immediacy of HT were developed by the Interagency Registry for Mechanically Assisted Circulatory Support (INTERMACS), which archives patients who receive durable Food and Drug Administration-approved mechanical circulatory support (MCS) devices (e.g., intra-aortic balloon pumps, extracorporeal membrane oxygenation (ECMO), Impella, TandemHeart) for the treatment of advanced heart failure. In 2009, INTERMACS developed profiles to integrate ESHF symptom severity with additional factors like hemodynamic instability and the use of MCS devices to prioritize the immediacy of HT (see Table 1 and Table 2) [7]. Patients with an INTERMACS profile of 1 or 2, which both include a deteriorating clinical status despite therapeutic inotropy, are often evaluated urgently for MCS device implantation and/or HT. These classifications guide therapeutic timing and establish the necessity for temporary and durable MCS as a bridge to transplantation, a bridge to recovery/decision, or destination therapy. Careful recipient evaluation is vital, as the use of MCS is associated with an increased risk of morbidity and mortality over time. For example, in 2015, INTERMACS data showed that patients treated with a continuous-flow device such as left ventricular assist devices (LVADs) to treat ESHF with reduced ejection fraction were associated with a 1- and 2-year survival of 80% and 70%, respectively [8]. However, while freedom from major non-cardiac adverse events did not improve among MCS survivors, their quality of life appeared to markedly improve throughout 24 months of therapy.

Another well-established system that assesses HT candidacy among ESHF patients was developed by the U.S. Organ Procurement and Transplantation Network (OPTN), an affiliate of the United Network for Organ Sharing (UNOS) (see Table 3) [9]. In 2018, the OPTN/UNOS expanded their previous stratification system from three to six “status” tiers with the addition of several objective factors, such as MCS recipients who experienced complications that included ventricular arrhythmia and infection. These changes prioritize critically ill patients who require temporary MCS or continuous therapeutic inotropy, with the greatest emphasis on those who experienced a ventricular arrhythmia and required ECMO. While the goal of the new system was to reduce waitlist mortality, some studies have scrutinized its efficacy. One of these was a study by Jawitz et al. that compared the short- and long-term survival (90 and 180 days, respectively) of HT candidates assigned to either the previous or new OPTN/UNOS systems, finding that post-HT outcomes between these groups were comparable [9]. However, candidates assigned to the new system were more likely to receive a bridge to transplant with temporary MCS devices instead of durable LVADs and experienced longer graft ischemic times. Ashraf et al. later confirmed comparable post-operative survival between the two models, but also demonstrated that the new screening policy reduced waitlist times (9.8% of HT candidates compared to 36.7% and 34.3%, *p* < 0.001) and the 1-year cumulative incidence of waitlist mortality among adults with congenital heart disease who received an orthotopic HT [10].

Non-cardiac end-organ function plays a decisive role when evaluating HT candidacy. Irreversible renal dysfunction, hepatic failure, or advanced pulmonary vascular disease are considered absolute contraindications to HT as they significantly elevate the risk of peri-operative mortality. Among predictors of poor outcomes, renal insufficiency and pre-operative ventilator dependence are particularly associated with diminished short- and long-term survival [11]. The presence of certain illnesses also presents a challenge, with malignancy considered an absolute contraindication, while systemic lupus erythematosus (SLE) is designated as a relative contraindication to HT but is possible under limited circumstances with immunotherapy [12]. Prior medication exposure also influences HT candidacy, with pre-transplant amiodarone use associated with higher rates of early graft dysfunction and increased 30-day and 1-year mortality [13].

Psychosocial evaluation is a mainstay of HT candidate selection, assessing behavioral, emotional, and social factors that can significantly influence post-transplant outcomes to optimize patient readiness, identify modifiable risk factors, and ensure equitable allocation of scarce donor organs. Psychiatric morbidity is highly prevalent in the pre- and post-operative HT population. For example, studies demonstrate that major depression is associated with worse pain outcomes and decreased quality of life among HT recipients, even after adjusting for confounding variables including pharmacological treatment and immunosuppressive therapy [14]. Depression, anxiety, and demoralization not only affect patient well-being but also correlate with medication nonadherence, a critical determinant of graft survival [15]. Traditional diagnostic frameworks like the DSM-IV may under-recognize these conditions, while broader tools like the Diagnostic Criteria for Psychosomatic Research (DCPR) capture additional syndromes, such as alexithymia (also known as emotional blindness) and type A behaviors (like impatience and hostility) that impact recovery and adjustment [16].

Structured psychosocial instruments have been developed to guide the evaluation process. The Stanford Integrated Psychosocial Assessment for Transplantation (SIPAT) is widely employed to provide a standardized risk assessment of transplant candidates, incorporating domains such as patient readiness, social support, psychological stability, and substance use (see Table 4). Candidates are scored as either excellent (0–6; recommend listing for transplant), good (7–20; list with minor monitoring of psychosocial risk factors), minimally acceptable (21–39; list under certain conditions while addressing risk factors), poor (40–69; defer listing while risk factors are addressed), or high risk (>70; transplant is not recommended due to risk factors) [17]. Studies have shown the effectiveness of the SIPAT as a screening tool, with a score lower than “Excellent/Good” among transplant candidates associated with greater nonadherence to maintaining follow-up clinic visits (*p* = 0.004), while also identifying modifiable psychosocial risk factors to pre-operatively optimize [11,18]. SIPAT is also a valuable post-operative instrument, with a national cohort study conducted in Ireland finding that 19% of transplant candidates previously labeled as excellent or good were later diagnosed with a post-operative psychiatric condition [19].

Despite the benefits of the SIPAT and other psychosocial assessments, its implementation remains variable across institutions. Historically, screening and long-term psychosocial evaluation in the HT population were low and inconsistent despite research indicating their importance [20]. According to the 2018 ISHLT guidelines regarding HT and LVAD implantation evaluation, while initial psychosocial evaluation followed by regular interval assessments is recommended, the timing between them was not specified and is ultimately dependent on the observations of a multidisciplinary team [21]. Due to the improved medication adherence, quality of life, and clinical outcomes associated with pre- and post-operative psychosocial monitoring and care, the integration of mental health professionals into multidisciplinary transplant teams is another recommendation [22]. However, explicit data on the effects of psychiatric pharmacological and non-pharmacological interventions on patient outcomes is limited and variable in quality, with possible directions into future research including formal studies to recommend the timing between psychosocial evaluations and strengthening of the evidence on psychiatric intervention among HT candidates as well as recipients [15].

### 2.2. Donor Selection and Recipient Allocation

Critical factors to consider in the ideal HT donor that affect later outcomes in post-operative recipients include heart size, ischemic time, and differences in donor-to-recipient demographics [23]. Heart size matching is essential to ensure adequate CO and avoid complications like right HF or graft dysfunction, with predicted heart mass (PHM) calculations increasingly used to optimize donor–recipient size matching, especially when there are significant body size discrepancies. Recently, the predicted heart mass ratio (PHMr), which accounts for height, weight, age, and gender, has been supported as the most reliable metric. It is calculated based on an equation developed by the Multi-Ethnic Study of Atherosclerosis (MESA), where the sum of the predicted masses of the left and right ventricle is determined, then stratified into either an undersized, matched, or oversized group. In a retrospective study analyzing the development of cardiomyopathies based on the PMHr scores of heart transplant patients, the incidence of valvular cardiomyopathies was 2% to 3% [24]. However, significant variability remains when comparing prognostic survival rates in patients who developed this form of cardiomyopathy compared to alternative forms, including hypertrophic or restrictive cardiomyopathy. Ischemic time, or the duration from donor heart cross-clamp to reperfusion in the recipient, also influences graft function and survival, with a median ischemic time of 3.2 h or more associated with primary graft dysfunction (PGD) [25].

One major factor in donor-to-recipient matching that has been previously studied is the impact of age. In one multicenter study conducted by Bergenfeldt et al., the age of donors and recipients was analyzed alongside other variables, including chronic illness (e.g., hypertension and diabetes mellitus), hospitalization, and MCS use, to determine its relationship with mortality [26]. The percentage of chronic illness, number of female donors, and those who expired from cerebrovascular incidents increased with donor age (age ≥ 25 years), while expiration due to trauma was associated more with younger donors (age < 25 years). Younger recipients (age < 38 years) demonstrated a greater association with non-ischemic cardiomyopathy and the use of MCS devices (e.g., ECMO, LVAD), while ischemic cardiomyopathy as well as chronic illness increased with recipient age (age ≥ 38 years). The resulting multivariable adjusted 1-year hazard ratio (HR) for mortality based on donor age was 1.16, which decreased to 1.06 at >10 years, while the 1-year HR for mortality based on recipient age increased from 1.05 at 1 year to 1.14 and 1.32 at 5–10 and >10 years, respectively, though the 1–3 year HR was 0.89 and the 3–5 year HR was not statistically significant. Several prior studies comparing donor and recipient age to mortality reinforced these findings, with an increase in donor and recipient age associated with increased mortality. Overall, donor age is an important predictor of mortality that should be considered with the recipient’s age to determine the likelihood of a successful cardiac allograft as well as the possible necessity for re-transplantation.

The sex of donors and recipients is an important consideration in cardiac allograft matching. Bergenfeldt et al. showed that the percentage of female donors increased over time, while female recipients decreased with age [26]. When compared to male recipients, female recipients also had an odds ratio of 1.19 for 1-year mortality when calculated using the index for mortality prediction after cardiac transplantation (IMPACT) score. A later single-center study by Breathett et al. compared the expected CV death of HT recipients (based on their frequency, as well as re-transplantation, according to UNOS criteria) to the observed CV death based on donor age and sex [27]. While a donor age of ≥40 years was associated with a significantly higher likelihood of mortality (*p* < 0.05) compared to a donor age of <40, recipients of a female-donor HT experienced a higher likelihood of mortality (*p* < 0.05), particularly the female recipients (*p* < 0.05). However, donor–recipient sex mismatches showed no significance for any combination (*p* < 0.62). While the authors highlighted the study’s small population size (e.g., ages were recorded for 706 of the 1294 adult recipients) and limited power, its findings align with other similar studies, with female-to-male allograft mismatching as successful as male-to-male matching when its size was equivalent [28]. One explanation for the higher mortality among female recipients compared to male ones is the pro-inflammatory effect of estradiol, which may increase the likelihood of autoimmune rejection, while testosterone is considered anti-inflammatory [29]. Regardless of etiology, female recipients are likely to benefit from greater post-operative observation to reduce HT-associated complications. Given that only 25% of all recipients are female, an increased rate of HT in this population with a greater number of studies into post-operative complications and management would also be beneficial [28].

Due to the peri- and post-operative requirements, as well as the complications associated with HT, prospective recipients undergo a comprehensive evaluation that incorporates comorbidities and psychosocial factors and screening for surgical contraindications to foresee which candidates are most likely to experience its post-operative benefits [18]. After this careful evaluation, these HT candidates can be further stratified to maintain optimal propriety in later graft allocation. Prognostic models like the Heart Failure Survival Score (HFSS), Seattle Heart Failure Model (SHFM), Meta-Analysis Global Group in Chronic (MAGGIC), and the Metabolic Exercise Test Data combined with Cardiac and Kidney Indexes (MECKI) are further attempts at refining risk assessments of ESHF patients to anticipate waitlist and post-transplant outcomes [19,30]. These predictive models combine qualitative (e.g., NYHA class, INTERMACS profiles) with quantitative data, including cardiopulmonary exercise testing (peak VO_2_ < 12 mL/kg/min) as well as invasive hemodynamic parameters such as elevated filling pressures and reduced cardiac index.

The HFSS was developed in 1997 as a prognostic model to score the likelihood of 1-year mortality [31]. It is composed of a non-invasive version that is calculated with a formula that includes mean arterial blood pressure, left ventricular ejection fraction (EF), and intraventricular conduction delay > 120 ms, as well as an invasive version wherein the pulmonary capillary wedge pressure is measured via right heart catheterization (RHC) and incorporated into the above variables when calculating the final score. The HFSS classifies candidates into one of three stratified groups that consist of a low- (≥8.1), medium- (7.2–8.09), and high-risk (≤7.19) cohort, with high-risk candidates prioritized for HT due to a greater likelihood of post-operative mortality during the first year. Due to a lack of significant difference in scoring between the non-invasive and invasive HFSS models, the non-invasive version is used more frequently. Ultimately, the HFSS has limited prognostic power as it was (1) previously shown by Zugck et al [31]. to be superior to a two-variable model consisting of LVEF and peak VO_2_, and (2) created before the true efficacy of modern HF therapies [e.g., beta-blockers, implantable cardioverter-defibrillator (ICD), and cardiac resynchronization therapy (CRT)] was taken into account [31,32]. To account for the absence of these factors, the SHFM was developed in 2006 and later modified in 2013 to incorporate 20 clinical factors, including demographics, laboratory measurements (e.g., BNP), HF management and device utilization [33,34]. The SHFM estimates 1-, 2-, and 3-year survival, can account for the therapeutic benefits of conservative and/or MCS device interventions, and is often calculated along with the HFSS, though its numerous variables make it difficult to be routinely employed, particularly in a clinical setting.

In 2013, a relatively condensed alternative to the HFSS was introduced in the MAGGIC risk score, which uses 13 clinical variables like BMI, current cigarette smoking and the presence of COPD to assess the 1- and 3-year mortality of HT candidates [35]. Unlike the SHFM, the MAGGIC risk score contains fewer variables that make it a more practical and rapid tool to screen a broader population, with a median score of ≥28 (range 27–30, *p* < 0.001) associated with a high risk of mortality [36]. Despite these advantages, the SHFM is considered better for evaluating HT candidates due to its greater number of factors, though some studies suggest that the MAGGIC score overestimates mortality risk while the SHFM underestimates it [37,38]. Another popular prognostic tool developed by Agostoni et al. and separate from the MAGGIC risk score is the MECKI, which assesses six variables such as kidney function [Modification of Diet in Renal Disease equation (MDRD)], percentage of predicted peak oxygen consumption (ppVO_2_), and minute ventilation compared to carbon dioxide production (VE/VCO_2_) [39]. When compared to the HFSS and MAGGIC risk score, a later study by the developers suggested that the MECKI provided superior prognostic power among HF patients, particularly those with reduced EF at the 2- and 4-year follow-up periods [40]. Despite this, the limitations of the MECKI include its tendency to overestimate mortality risk, its relatively recent validation (2023) and a lack of validation studies among a broad and diverse U.S. cohort [41]. While these prognostic tools offer a useful objective guide in the allotment of donor hearts, their absolute effectiveness remains a point of contention. Because of this, the International Society for Heart and Lung Transplant (ISHLT) updated the 2024 guidelines to prioritize recipients with a 1-year survival <85%, while not using the scores of any predictive screening method as the sole determinant for HT [30].

### 2.3. Quality-of-Life Considerations

The impact of HT on health-related quality of life (HRQoL), which refers to physical as well as psychosocial characteristics that include functional capacity, pain intensity, and emotional well-being, is highly influential in guiding donor organ allocation and medical therapy. As previously discussed, ESHF patients who received HT experienced improved mortality, with 53% of recipients living 10 years or more post-transplant [4]. Moreover, a systematic review from January 2020 to December 2022 found that HT led to significantly favorable changes in a majority of HRQoL parameters, with some studies that indicated continued benefit after 20 years [42]. Because of this, there are many questionnaires to assess HRQoL in HT candidates to predict the likelihood of mortality and recovery. The most widely used surveys include the WHOQoL-BREF [43], the Short-Form 36 (SF-36), the Kansas City Cardiomyopathy Questionnaire (KCCQ), and the Minnesota Living with Heart Failure Questionnaire (MLHFQ) [43,44]. Among these questionnaires, the KCCQ and MLHFQ are often referenced and preferred for pre- and post-HT surveillance and are often performed simultaneously, though the KCCQ is often preferred for its superior monitoring of symptoms and functional status among HF patients with preserved EF [28,45]. Overall, the ISHLT recommends the regular assessment of HRQoL among HT candidates and recipients, while a specific questionnaire to do so has not been endorsed.

### 2.4. Decision Pathway: LVAD as a Bridge to Transplant or Destination Therapy

For those with ESHF who may either qualify or are ineligible for HT, the limited number of available cardiac donors makes life-prolonging alternatives a necessity. Continuous-flow LVADs (CF-LVADs) are a frequent treatment option associated with significant complications that must be considered if implemented as a bridge to transplant (BTT) for HT or as a destination therapy. Although LVAD recipients experience a similar rate of survival when compared to the recipients of temporary marginal donor hearts (defined by donors aged 55 years or older, hepatitis C-positive donors, prior or current cocaine users, and donors with EF < 45% or a donor-to-recipient BMI mismatch > 20%), they are also at an increased risk of bleeding, infection, stroke, and/or right-sided HF [46,47]. For example, one comparative study between the HeartMate II CF-LVAD and other devices showed a significant number of post-operative bleeding events in the experimental (44.4%) as well as the comparison groups (38.5%) [48]. In terms of infection among LVAD recipients, one clinical trial showed an increased risk of infection after HT, while a later observational study did not show a significantly higher risk of post-operative infection, CAR, or mortality [49,50]. Another important complication is stroke, with a 2-year randomized control trial showing a 23.1% occurrence among centrifugal CF-LVAD recipients, while differences in atrial fibrillation, anticoagulation regimens, and blood pressure did not significantly correlate with the rate of stroke [51]. Another complication observed in the trial included right ventricular (RV) failure, with a prevalence of 34% in HeartMate III recipients compared to 4.1% who received an RVAD.

### 2.5. Pre-Operative Surveillance of Heart Transplantation Candidates

During the pre-operative period, it is recommended that HT candidates awaiting a cardiac allograft receive routine follow-up to determine the presence of any new and/or worsening concerns that may warrant earlier prioritization [30]. While inter-organization guidelines previously recommended regular laboratory assessment and physical examination, the explicit intervals for follow-up by the ISHLT are recommended for only several conditions, including cancer screening, as well as human leukocyte antigen (HLA) antibody and RHC screening every 3 to 6 months.

The ISHLT recommends routine HLA antibody testing for all waitlisted HT candidates to screen for sensitization [30]. Sensitization is an immune state when a patient has circulating serum HLA antibodies that increase the likelihood of CAR, particularly HLA-DR mismatches, and is associated with inferior survival outcomes [52]. Sensitization, as measured by panel reactive antibodies (PRAs), is a significant risk factor for graft failure, especially in patients with prior mechanical circulatory support [53]. However, challenges exist when utilizing HLA matching in ESHF patients. In a retrospective analysis of over 33,000 adult HT recipients from the UNOS database (2005–2021), while a high degree of HLA mismatching was associated with increased acute rejection, it was not a significant predictor of overall survival [24]. Because of this, along with time constraints, HLA is not a strict criterion for donor-to-recipient cardiac allograft selection and is instead screened for prior to HT for retrospective study and for possible donor-specific antibody identification afterward [30]. Mismatching at the HLA-DR locus is also associated with an increased risk of pathologic immune-mediated remodeling of the vasculature, known as cardiac allograft vasculopathy (CAV), suggesting it as a potential focus for further study.

Routine RHC is recommended in waitlisted HT candidates to optimize medical therapy, define HT candidate urgency, assess end-organ injury, and guide the use of temporary and durable MCS implantation [30]. One additional use for RHC that has been increasingly studied is measurement of the right ventricular–pulmonary artery (RV-PA) coupling ratio. The RV-PA coupling ratio indicates the relationship between the right ventricle’s contractility against resistance from pulmonary afterload, with prior studies showing a ratio of <0.36 mm/mmHg (also known as uncoupling) is associated with higher mortality among geriatric heart failure patients [54,55]. While RV-PA coupling is often collected via RHC, it can also be approximated via non-invasive echocardiography by comparing TAPSE to pulmonary artery systolic pressure (PASP). For example, a retrospective study by Acar et al. showed that a higher TAPSE/PASP was strongly associated with exercise capacity (measured as peak VO_2_) and a lower risk of adverse events, including all-cause mortality [56]. However, known limitations of TAPSE/PASP include a complete reliance on measurable tricuspid regurgitation to estimate PASP, potential errors due to severe valve disease, and sensitivity to fluid volume instead of right ventricular contractility. Even so, its non-invasive utility and the possible prognostic advantages of echocardiographic surveillance as an adjunct to RHC and/or cardiopulmonary exercise testing during the pre-operative phase for HT candidates may warrant further studies and validity.

Another relevant cardiac variable that RHC and echocardiography can measure is elevated pulmonary vascular resistance (PVR), which is associated with a reduced responsiveness to vasodilators and inotropic agents, as well as higher rates of post-operative right heart failure and mortality [11,57,58]. However, a later cross-sectional study by Stein et al. showed that Doppler echocardiography revealed similar findings when compared to RHC, thus providing another possible measurement method for regular hemodynamic surveillance among HT candidates while reducing the frequency of invasive procedures [59]. Kuppahally et al. later reaffirmed the reliability of echocardiography when measuring multiple hemodynamic variables, including PVR, yet borderline PVR measurements were less definitive and required a subsequent RHC [60].

Additional research by Crawford et al. suggested that a PVR ≥ 3 WUs is associated with an increased 30-day mortality, even in HT patients with a mean pulmonary artery pressure < 25 mmHg [61]. In 2022, Rieth et al. conducted a study of 333 HT patients, which found that the traditional cutoff of PVR ≥ 3 WUs did not show a statistically significant association with mortality, whereas a lowered cutoff of PVR ≥ 2.2 WUs showed slightly greater significance (*p* = 0.04). The study also showed greater rates of post-transplant mortality and right HF among patients with higher pulmonary arterial elastance (E_a_) (*p* < 0.0001), a measurement of the total right ventricular afterload, which is affected by pulmonary arterial compliance and is a calculated ratio of end-systolic pressure to stroke volume (E_a_ ≈ P_es_/SV). An E_a_ > 1.45 exhibited a 12-month mortality of 47% and an overall mortality of 59%, while mortality among those with an E_a_ < 1.45 was significantly reduced (log-rank HR 0.38, 95% CI 0.19–0.76; *p* < 0.0001). Of note, Alnsasra et al. recently observed 2169 HT recipients who were bridged with LVAD, finding that patients who were given sodium nitroprusside with a later successful reduction in PVR to less than 2.5 WUs experienced a lower peri-operative mortality compared to those who were unsuccessfully reduced (40.6% and 27.5%, respectively) [62]. Overall, while PVR has been shown to be a moderately reliable predictor of mortality among prospective HT recipients, a method that incorporates a formal cutoff along with other significant measurements like E_a_ can serve as a valuable tool to determine HF severity, as well as to predict post-operative mortality and guide graft allocation.

**Figure 2:**
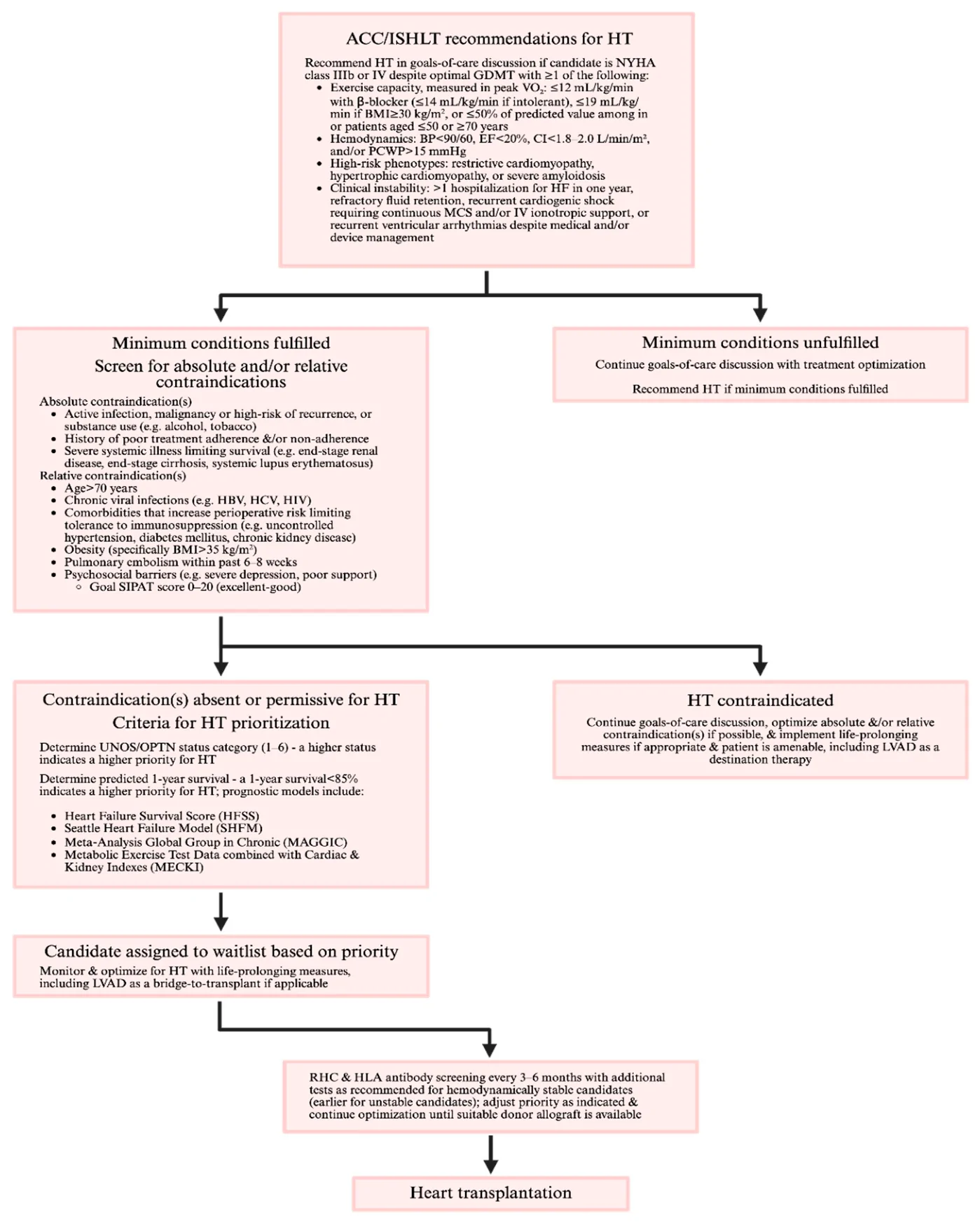
Pre-transplant stage of heart transplantation, illustrating an approximation of the pre-transplant stage of HT, including the initial appraisal of candidates based on ACC/ISHLT recommendations, screening for contraindications, prioritization, observation prior to surgical intervention, and alternative pathways if the minimum conditions for HT are not fulfilled or HT is contraindicated. Abbreviation(s): HT, heart transplantation; ACC, American College of Cardiology; ISHLT, International Society of Heart and Lung Transplantation; peak VO_2_, peak oxygen uptake; BP, blood pressure; EF, ejection fraction; CI, cardiac index; PCWP, pulmonary capillary wedge pressure; HF, heart failure; MCS, mechanical circulatory support; IV, intravenous; SIPAT, Stanford Integrated Psychosocial Assessment for Transplantation; LVAD, left ventricular assist device. Created in BioRender. Ringor, M. (2026). https://BioRender.com/03rdye1 (accessed on 25 August 2026).

Overall, the pre-transplant stage necessitates an intricate and individualized screening process to select and then optimize HT candidates for the most ideal survival outcomes [Figure 2].

## 3. Peri-Transplant Stage (Transplant Therapies)

### 3.1. Goals of Peri-Operative Management

Hemodynamic instability in the peri-operative period is common immediately after cardiac surgery and usually multifactorial, with causes that include bleeding, primary and secondary graft dysfunction, arrhythmia, vasoplegia, pulmonary hypertension, and a systemic inflammatory response (SIRS) [63]. To avoid hemodynamic compromise and promote recovery, the preload, contractility, and afterload of the HT recipient must achieve an ideal cardiac output (CO). This includes adequate fluid resuscitation to maintain adequate preload, inotropes to support the contractility of a labile myocardium, and vasopressors to increase afterload and maintain a normal mean arterial pressure (MAP).

Patients are intubated and placed on mechanical ventilation before transfer to the cardiac surgery intensive care unit (ICU), and the aforementioned fluid resuscitation, inotropes and vasopressor support, along with continuous hemodynamic monitoring based on the guidelines published by the ISHLT, are carried out [64]. Such monitoring devices include an arterial line to measure blood pressure, as well as a Swan–Ganz pulmonary artery (PA) catheter to monitor PA pressures, central venous pressures (CVP), and CO. Other non-invasive measures include electrocardiographic monitoring of the heart rate and rhythm, oxygen saturation, end-tidal carbon dioxide, and urine output with a Foley catheter. The three crucial variables to observe in the peri-operative stage that impact hemodynamic status include the volume status or preload, afterload or the resistance, and the contractility of the heart.

### 3.2. Immediate Post-Operative Care

ICU protocols for HT recipients in the immediate post-operative period, as well as the risk of RV failure, PGD, and vasoplegia, require hemodynamic optimization, early detection and management of PGD, immunosuppression initiation, infection prophylaxis, and multidisciplinary care. The American Society of Transplantation emphasizes the need for coordinated, multidisciplinary ICU management involving intensivists, transplant cardiologists, surgeons, pharmacists, and other specialists to address the complex needs of these patients [65].

Frequent complications that adversely affect hemodynamic stability and that occur during recovery after HT include, but are not limited to, respiratory failure, renal failure, fluid overload, and infection [66]. Cardiac function can be assessed via renal function and volume status, with an increased risk for acute kidney injury (AKI) once CVPs are too high for optimal Starling cardiac performance. This elevated right-sided cardiac pressure creates an elevated renal venous pressure, which reduces renal perfusion that can lead to AKI [63]. Additionally, fluid overload increases the risk for peripheral edema and impaired oxygenation, leading to respiratory failure and/or compartment syndrome.

According to ISHLT guidelines, HT recipients require vasoactive support in the immediate post-operative period, with one or multiple various inotropes (e.g., epinephrine, isoproterenol, milrinone, and dobutamine) employed to support the newly implanted heart [64,66]. Continuous inotropic support for 3–5 days is recommended to achieve hemodynamic stability for the denervated, transplanted heart. Inotropic and vasopressor support is titrated to maintain adequate CO and end-organ perfusion, while pulmonary hypertension and ventilatory status are optimized to prevent RV failure [65]. In most grafts, the resting heart rate is typically anywhere between 90 and 110 bpm, but tachycardic responses to stress and other stimuli are blunted due to the loss of parasympathetic tone. Permanent pacemaker implantation is thus considered in cases of persistent sinus nodal dysfunction and bradycardia.

In order to optimize renal function, control volume status and avoid hypoperfusion, it is vital to maintain an MAP of at least 65 to 70 mmHg, as well as a CVP of 5 to 12 mmHg [64]. For HT recipients who experience hypervolemia and elevated filling pressures, diuresis and/or renal replacement therapy are initiated to remove volume, reduce right-sided venous congestion, and improve cardiac as well as renal function. Due to the initiation of immune suppression in the peri-operative period and its nephrotoxic potential, careful management is necessary to prevent AKI. Because of this, the overall care of HT recipients should be monitored by a multidisciplinary team, which also includes a pharmacist to observe levels of creatinine and immunosuppression to adjust therapy as needed.

In regard to ventilator management, goals include the avoidance of hypoxemia, hypercarbia, and acidosis, which all may worsen PVR. Oxygen should be titrated to the lowest level needed to maintain adequate oxygen saturation, as hyperoxia may cause coronary vasoconstriction, impairment of diastolic relaxation, and increased myocardial injury [67]. Increased mortality has been witnessed in critically ill patients with a PaO_2_ > 150 mmHg. When planning for extubation, the standard criteria for consideration entail adequate mental status, gas exchange, respiratory muscle strength, and hemodynamic stability.

One particular challenge to extubation is RV dysfunction, as positive end-expiratory pressure can mitigate alveolar collapse as well as hypoxia and improve RV afterload by reducing hypoxic pulmonary vasoconstriction [65,67]. However, withdrawal of these benefits may instigate RV dysfunction and worsen hemodynamic instability if not monitored and promptly managed. To manage observed RV dysfunction after extubation, strategies include inhaled nitric oxide or prostacyclins to reduce PVR, strict fluid resuscitation to avoid volume overload, and the use of supplemental oxygen as needed to prevent hypoxia, hypercarbia, and/or acidosis that can increase pulmonary artery pressure.

## 4. Post-Transplant Stage (Post-Therapies)

### 4.1. Immunosuppressive Therapy

#### Induction and Maintenance

Following heart transplantation (HT), about 50% of recipients receive a short course of induction therapy (IT) immediately to prevent early CAR and allograft dysfunction for high-risk recipients [68]. However, the strategy of IT remains controversial, with many HT centers creating their own regimens that each carry the risk of significant complications, including infection, anaphylaxis and cytokine release syndrome (CRS). In essence, the goal of IT is to achieve early immunosuppression, paving the way for the later addition of calcineurin inhibitors to avoid their nephrotoxic effects and facilitate early tapering of glucocorticoids [69]. In current practice, about 30% of HT programs utilize interleukin-2 receptor antagonists (IL2RA) such as basiliximab, and 20% deploy polyclonal anti-thymocyte globulin (pATG), while other IT agents such as morumonab-CD3 have been discontinued in favor of safer alternatives due to higher complication rates, which include CRS and anaphylaxis [70]. For example, the Cedars–Sinai Heart Transplant Program favors pATG for its improved efficacy and reduced risk of antibody production, administering 1.5 mg/kg daily for 5 days immediately after HT [71]. However, highly sensitized patients are given acetaminophen, diphenhydramine, and methylprednisolone prior to receiving pATG to reduce the likelihood of complications that include CRS and anaphylaxis.

Several prior studies comparing IL2RA and pATG showed inconclusive evidence on the efficacy of IT. For example, a systematic review and meta-analysis by Kugathasan et al. reviewed multiple IT observational studies and found with low certainty that pATG may decrease 5-year CAV (OR 0.82) compared to no IT, as well as 30-day (OR 0.85), 1-year (OR 0.87), and overall (HR 0.84) mortality compared to basiliximab [72]. On the other hand, it was suggested with very low certainty that basiliximab may increase 30-day (OR 1.13) and 1-year (OR 1.11) mortality compared to no IT. The remainder of findings comparing basiliximab, pATG, and no IT were inconclusive with very low certainty due to imprecision and biases attributed to improper randomization. Another meta-analysis by Briasoulis et al. found that IL2RA and pATG had similar rates of all-cause mortality, infection, and malignancy when compared to no IT, though pATG had lower rates of moderate–severe rejection compared to IL2RA among the case–control trials reviewed [69]. One group who appeared to benefit from IT was HT recipients who received MCS including ECMO, with worse survival among those who did not receive it (*p* < 0.0001) [73]. Overall, appraisal of the current data regarding IT suggests benefit among those at high risk of post-HT all-cause mortality, though further determination of whether the remainder of recipients may benefit from it may require more precise clinical trials.

Similar to IT, maintenance therapy (MT) is another critical part of maintaining the integrity of the graft. Unlike IT, MT is a lifelong regimen that includes an antimetabolite (mycophenolate), a calcineurin inhibitor (tacrolimus or cyclosporine), and glucocorticoids (prednisone) [74,75]. Due to the side effects of these medications, namely calcineurin inhibitors and glucocorticoids, it is recommended to slowly taper and reduce the target levels of these medications (see Table 5). For example, prednisone is tapered from higher doses down to 5 mg daily in 6–12 months post-transplantation [74]. Calcineurin inhibitors are notorious for their nephrotoxicity, so other immunosuppressive agents such as mTOR inhibitors (sirolimus, everolimus) have been looked at as potential replacements [76]. The MANDELA Trial compared everolimus with mycophenolate, following a discontinuation of cyclosporine to reduced-dose cyclosporine with everolimus. At the end of the 18-month period, the everolimus-mycophenolate group saw improved renal function after cyclosporine was discontinued, at the cost of higher rates of biopsy-proven rejection, although without hemodynamic instability [77]. The SCHEDULE Trial conducted by Arora et al. also compared outcomes with a similar methodology as the MANDELA Trial, but followed their participants over a 3-year period [78]. In this study, HT recipients who received post-operative low-dose everolimus, low-dose cyclosporine, mycophenolate mofetil and corticosteroids, followed by cyclosporine discontinuation and full-dose everolimus at 7–11 weeks, were associated with a reduced progression of CAV at 12 and 36 months compared to those who received standardized cyclosporine immunosuppression. Gastafsson et al. conducted a follow-up of trial participants at 5 and 7 years, with better renal function, as well as lower rates of CAV, observed in the treatment group compared to the control group (53% versus 74%, respectively; *p* = 0.037), while the incidence of biopsy-proven acute rejection was higher in the former but without hemodynamic instability (*p* = 0.23) [79]. Another potential replacement for calcineurin inhibitors is anti-CD38 antibodies such as daratumumab. The latter is used in kidney transplantation to treat antibody-mediated rejection but there is scant data from case reports as to its efficacy among HT recipients [80,81].

### 4.2. Management of Short- and Long-Term Complications

#### 4.2.1. Acute Cellular Rejection (ACR)

Acute cellular rejection (ACR) is a T cell-mediated mechanism in which recipient immune cells recognize a donor-derived antigen and subsequently mount an immediate attack against it, with as many as 24% of HT recipients experiencing one episode within the first year [67]. The recognition of the donor-derived antigen, also known as allorecognition, occurs when major and/or minor histocompatibility antigens from a donor act as alloantigens to activate a recipient’s alloimmunity in one of two pathways. In the direct pathway, donor-derived dendritic cells migrate from a transplanted organ to the recipient’s secondary lymphoid tissues, where they present intact donor alloantigens to recipient T cells, thereby initiating an immune response. In the indirect pathway, recipient dendritic cells process graft-derived proteins and then present these donor peptides to recipient T cells. Other important mediators of ACR include interleukin-2, tumor necrosis factor-beta (TNF-β), and interferon-gamma (IFN-γ). Through these mechanisms, APCs activate cytotoxic T cells by stimulating clonal expansion, differentiation into effector cells, and migration to the donor-derived antigen, leading to tissue destruction [82].

Based on animal models, there is promising evidence for novel therapeutics to treat ACR, such as abatacept, which binds to CD80 and CD86, thereby blocking CD28 engagement and T cell activation. While rodent studies showed encouraging results, subsequent trials with non-human primates failed to achieve the anticipated induction of tolerance [82]. Another drug that is currently undergoing clinical trials is belatacept, with preliminary data suggesting that it may prevent chronic cellular rejection, though it was also associated with higher rates of early acute cellular rejection.

#### 4.2.2. Antibody-Mediated Rejection (AMR)

Antibody-mediated rejection (AMR) is a spectral disease process with varying severities and progressions, often mediated by B cells and anti-HLA antibodies, that can begin at any time post-transplantation, eventually causing the development of allograft dysfunction, damage and loss. AMR in HT is characterized by certain cardinal features, which include chronic vascular lesions that manifest as vasculopathies or arterioscleroses. A diagnosis of acute or chronic AMR is established when three criteria are met: (1) serologic evidence of donor-specific antibodies (DSAs), (2) C4d deposition within peritubular capillaries, and (3) histopathologic evidence of acute or chronic allograft injury.

One sensitive and highly specific biomarker of acute AMR is diffuse C4d positivity, defined as staining of more than 50% of peritubular capillaries with intensity greater than 1+ [83]. Anti-HLA DSAs are also well-characterized prognostic biomarkers that predict relevant clinical outcomes for different types of organ transplantation, including HT. However, several cohort studies demonstrated their absence in 40–60% of patients with histological features of AMR at the time of biopsy [84]. To address this anomaly, previously discovered non-HLA DSAs identified in this atypical population are currently undergoing study and validation to provide an additional tool for AMR detection [84,85]. Because of the varying degrees of pathogenicity between anti-HLA antibody subclasses, another surveillance approach is to perform an allograft biopsy to identify IgG subclasses as well as composition, titer levels, and their capacity to activate complement. These parameters can better guide the ability to predict AMR and transplant outcomes, including allograft loss.

The main goals of treatment for AMR are to eliminate circulating anti-HLA DSAs and either neutralize their activity, suppress their generation, or achieve both. The mainstays of therapy are plasma exchange, intravenous immune globulin (IVIG), and/or glucocorticoids. Evidence shows that the use of plasma exchange and IVIG improves short-term outcomes, although long-term outcomes remain poor. Other agents, such as protease inhibitors and anti-CD20 monoclonal antibodies, are under investigation for their potential to improve allograft survival by reducing the formation of anti-HLA DSAs, but have yet to demonstrate clinical benefit [85].

#### 4.2.3. Cardiac Allograft Vasculopathy (CAV)

Cardiac allograft vasculopathy (CAV) is a major cause of late graft loss and is described as diffuse, concentric narrowing of the coronary arteries, with characteristic findings including intimal fibromuscular hyperplasia, atherosclerosis, and vasculitis [86]. The incidence of CAV increases with time, developing in ∼30% of patients at 5 years and almost 50% at 10 years. Studies show a complex interplay between the non-immune and immune risk factors, which leads to endothelial injury and disease development. Non-immune risk factors for the development of CAV related to the organ donor include older age, brain death, and ischemia–reperfusion injury. Immune risk factors include the presence of alloantibodies and the occurrence of acute rejection.

The primary drivers for CAV include the host immune system and alloantigens. Interestingly, CAV lesions are noted to stop at the suture line between the donor and recipient tissue. The top five key mechanisms through which CAV develops include alloimmune T cell responses, donor-specific antibodies (DSAs), innate immune cell involvement, non-immune injury mediated by ischemia–reperfusion, and viral and metabolic factors [86]. Recipient T cells recognize donor HLA molecules and release cytokines such as IFN-γ and TGF-β, driving smooth muscle cell proliferation and intimal thickening. DSAs further contribute by binding to endothelial cells, activating complement, and amplifying vascular injury through recruitment of T cells and NK cells. Innate immune cells, including macrophages and NK cells, release growth factors and inflammatory mediators that enhance vascular remodeling, while peri-transplant ischemia–reperfusion injury primes the graft vasculature for immune attack. Additional factors such as CMV infection and hyperlipidemia exacerbate endothelial activation and immune responses, accelerating the progression of CAV.

Preventive measures such as preventing CMV infections, avoiding rejection, managing vascular risk factors, and using certain medications, such as statins and mTOR inhibitors, which slow the progression of the disease, are the mainstay of current therapeutic treatment for CAV [86]. Medications like mycophenolic acid (MPA) and mTOR inhibitors such as sirolimus and everolimus reduce the development and progression of CAV, and studies have proven that they reduce CAV incidence and disease when assessed by intracoronary vascular imaging. Other interventions include percutaneous revascularization for obstructive coronary artery disease and re-transplantation, which is typically a last resort, for allograft dysfunction. Risks associated with early post-transplant mTOR inhibitor administration include cytopenias, infections, effusions, increased nephrotoxicity, CAR when used in conjunction with calcineurin withdrawal, and decreased wound healing. In the EVERHEART trial, while there was no difference in the primary outcome of wound healing and efficacy, immediate everolimus administration induced more adverse events, including pericardial effusions, compared to delayed administration [87].

#### 4.2.4. Infection Prophylaxis

For patients on powerful immunosuppressives, measures to prevent opportunistic infections are a routine part of post-transplantation care. Infections account for 20% of deaths during the first year post-HT [88]. The common culprits of infection are cytomegalovirus (CMV), *Aspergillus* species, *Pneumocystis jiroveci* (PCP), and *Nocardia* species [89]. During the first 6–12 months, HT recipients will be started on valganciclovir for CMV prophylaxis, trimethoprim-sulfamethoxazole (TMP-SMX) for PCP prophylaxis, and fluconazole/itraconazole or amphotericin B for fungal infections in those deemed high risk for systemic fungemia.

### 4.3. Rejection Surveillance

#### 4.3.1. Endomyocardial Biopsy

Endomyocardial biopsy (EMB) is the gold standard for diagnosing tissue ACR, AMR and CAV, but is an invasive procedure that has complications such as tricuspid regurgitation, carotid perforation, and iatrogenic cardiac injury, leading to pericardial effusions [90,91]. Additionally, interobserver variability in grading rejection and the site of the biopsy are also factors that can hinder the diagnosis [92]. There is also a chance of false negatives, as the biopsy would have to be taken from the structure that is being rejected [93]. For example, a rejection occurrence in the left ventricle will not be detected by a biopsy of the right ventricle. Furthermore, in about 10% of patients with left ventricle systolic dysfunction, EMB fails to establish a diagnosis [94]. Recently, the routine use of EMB for rejection surveillance has been challenged given the low incidence of rejection in the modern era of immunosuppressives [90]. In 2023, the ISHLT released updated guidelines based on data from studies that graded the histology of EMB samples from no rejection (Grade 0) to moderate- (Grade 2R) or high-severity (Grade 3R) rejection [Table 6] [76]. During post-transplant surveillance, EMBs performed in asymptomatic patients prescribed calcineurin-inhibitor- and mycophenolate-based immunosuppressive regimens showed a 1% and 2% yield for detecting a Grade ≥ 2R, respectively, while the results among symptomatic patients were 18% in one study. Given these findings and the surge in non-invasive tests such as cell-free DNA and cardiac imaging, the role of EMB may be relegated to niche circumstances [95]. However, non-invasive tests have problems in their diagnostic accuracy, and there is a lack of protocols that incorporate these non-invasive tests into routine surveillance of HT recipients.

#### 4.3.2. Gene Expression Profiling

To avoid the invasiveness of an EMB, transplant centers nationwide have adopted additional non-invasive methods for regular surveillance. One such test is gene expression profiling (GEP), which emerged as one of the first serum tests for CAR. Even before its formal approval by the Food and Drug Administration in 2008, it had been used since 2005 as a non-invasive test for rejection surveillance [96]. GEP is a gene-profile test that looks at certain genes and gives a score based on the expression of the analyzed genes. GEP later became validated and viewed as a pioneer marker for non-invasive testing thanks to the Invasive Monitoring Attenuation Through Gene Expression (IMAGE) trial [97]. Published in 2010, this trial compared the efficacy of GEP to routine EMBs for CAR surveillance. Following a 19-month period, both interventions had similar rejection rates, with hemodynamic instability and graft dysfunction leading to death or re-transplantation for 14.5% and 15.3% of the GEP and EMB groups, respectively. In the GEP cohort, only 6 of the 34 rejection cases were detected by GEP alone, whereas the other cases were diagnosed based on clinical symptoms and overt signs of left ventricular systolic dysfunction on echocardiography. Nevertheless, the authors reported that the use of EMBs was significantly reduced in the GEP cohort, and patients in the non-invasive group were more satisfied with their care compared to the invasive surveillance strategy.

Additional concurrent studies, called the Cardiac Allograft Rejection Gene Expression Observational (CARGO) trials, further supported the findings of the IMAGE trial to discover a non-invasive alternative to CAR monitoring [98,99]. The CARGO I trial compared EMB with an 11-point GEP test to verify whether it could reliably identify the absence of CAR in stable, low-risk patients [98]. Based on a scoring system from 0 to 40 (rejection score ≥20), the results of the CARGO I trial showed that low molecular scores in the GEP test were associated with a very low risk of moderate-to-severe rejection at ≥1 year post-transplant and a high negative predictive value (NPV > 99%). The subsequent CARGO II trial further continued to compare EMB with GEP, with similar rates of rejection detected between both study groups from 2–6 months and >6 months post-HT [99]. Using a score cutoff of 34, GEP had a strong NPV of 98.4% but a poor positive predictive value of 2.6%. Currently, data from over 2000 HT recipients are being tracked by the Outcomes AlloMap Registry (OAR) study, which seeks to conduct long-term surveillance of GEP over 5 years and evaluate its outcomes [100]. As discussed later, donor-derived cell-free DNA (dd-cfDNA) is another non-invasive test. However, combining the two tests does not confer a synergistic effect and has similar outcomes compared to GEP alone [101].

#### 4.3.3. Donor-Derived Cell-Free DNA (dd-cfDNA)

A non-invasive alternative approach to heart transplant surveillance in patients after heart transplantation is percent donor-derived cell-free DNA (%dd-cfDNA), wherein patient sampling is analyzed for a threshold percentage to a known biomarker associated with cardiac allograft damage to detect acute rejection. As previously mentioned, while EMB remains the gold standard for diagnosing CAR, it retains issues including a significant probability for false negative results based on location as well as a 10% diagnostic failure rate in patients with left ventricle systolic dysfunction [94]. Because of this, more studies have employed percent dd-cfDNA (%dd-cfDNA) to detect acute cellular rejection and antibody-mediated rejection, with some indicating it 3.2 months earlier than EMB [102,103,104]. These studies vary in their analytical techniques to determine %dd-cfDNA, such as shotgun sequencing versus digital polymerase chain reaction (PCR). In terms of the latter, one observational study showed that the % sensitivity and specificity of dd-cfDNA for CAR were 0.76 and 0.83 compared to EMB, respectively, with an area under the curve (AUC) of 0.81, though omitting stable patients with pericardial effusions increased the AUC to 0.86 [105].

Other studies also analyzed multiple biomarkers, as well as changed the mode of analysis for %dd-cfDNA, to search for genes with greater association with AR while optimizing positive and negative predictive values [106,107]. In one study, combining %dd-cfDNA with N-terminal prohormone of brain natriuretic peptide (NT-proBNP) significantly increased the NPV to 98.1%, though it highlighted a shortcoming of the diagnostic accuracy of %dd-cfDNA, the threshold of a biomarker to determine rejection [108]. Because of its novelty, there are no protocols for how to measure %dd-cfDNA, as well as no knowledge of which biomarkers retain the highest degree of diagnostic accuracy [109]. Other studies presented differences in detection, with more episodes of antibody-mediated rejection found and treated compared to acute cellular rejection [110]. Despite these shortcomings, %dd-cfDNA remains a promising non-invasive candidate for post-transplant surveillance to detect AR, with a significantly robust amount of diagnostic accuracy that further research may improve over time.

#### 4.3.4. Brain Natriuretic Peptide

Additional objective measurements including traditional cardiac biomarkers such as brain natriuretic peptide (BNP) have been studied to determine their efficacy in detecting CAR after HT. For example, Vorlat et al. studied 105 post-mortem HT donors and found a very low risk of poor cardiac function with a BNP < 132 pg/mL, with a negative predictive value of 94%, while an elevated BNP above this threshold predicted poor cardiac outcomes with a sensitivity of 56% and a specificity of 86% [111]. Although an elevated BNP among donors was associated with increased 30-day mortality (r = 0.29, *p* = 0.0029), long-term survival was not, and no significant association between troponin and survival was shown. As a consequence, BNP is utilized as a screening tool during the post-HT observational period.

#### 4.3.5. Electrocardiogram

While endomyocardial biopsy is the gold standard of diagnosing CAR, non-invasive imaging techniques can be used to screen for signs of rejection. The electrocardiogram (EKG) is one such modality. Its main advantage is that it is cheap, fast, and ubiquitous. Following transplantation, EKG changes that affect the QT interval and interventricular conduction delays occur, both of which are caused by changes in sympathetic innervation of the allograft [112]. However, these changes are non-specific and are not evidence of developing rejection, with QT interval changes found to be similar between mild, moderate, and severe rejections [113]. In case studies, EKG findings such as right bundle branch block (RBBB), ST-segment elevations, and low QRS voltages were present at the time of rejection [114,115]. Similarly, atrial flutter or atrial fibrillation was also present during the rejection [115]. With the advancement of artificial intelligence (AI), computer models are being developed to analyze EKGs for evidence of CAR. In a small study, an AI model was able to predict ACR with 100% sensitivity and 60% specificity [112]. This model carries a 5% positive predictive value and a 100% negative predictive value. While EKG could be a useful tool in detecting early rejection, more studies and potentially more diagnostic models should be developed to better improve this diagnostic modality.

#### 4.3.6. Echocardiogram

**Figure 3:**
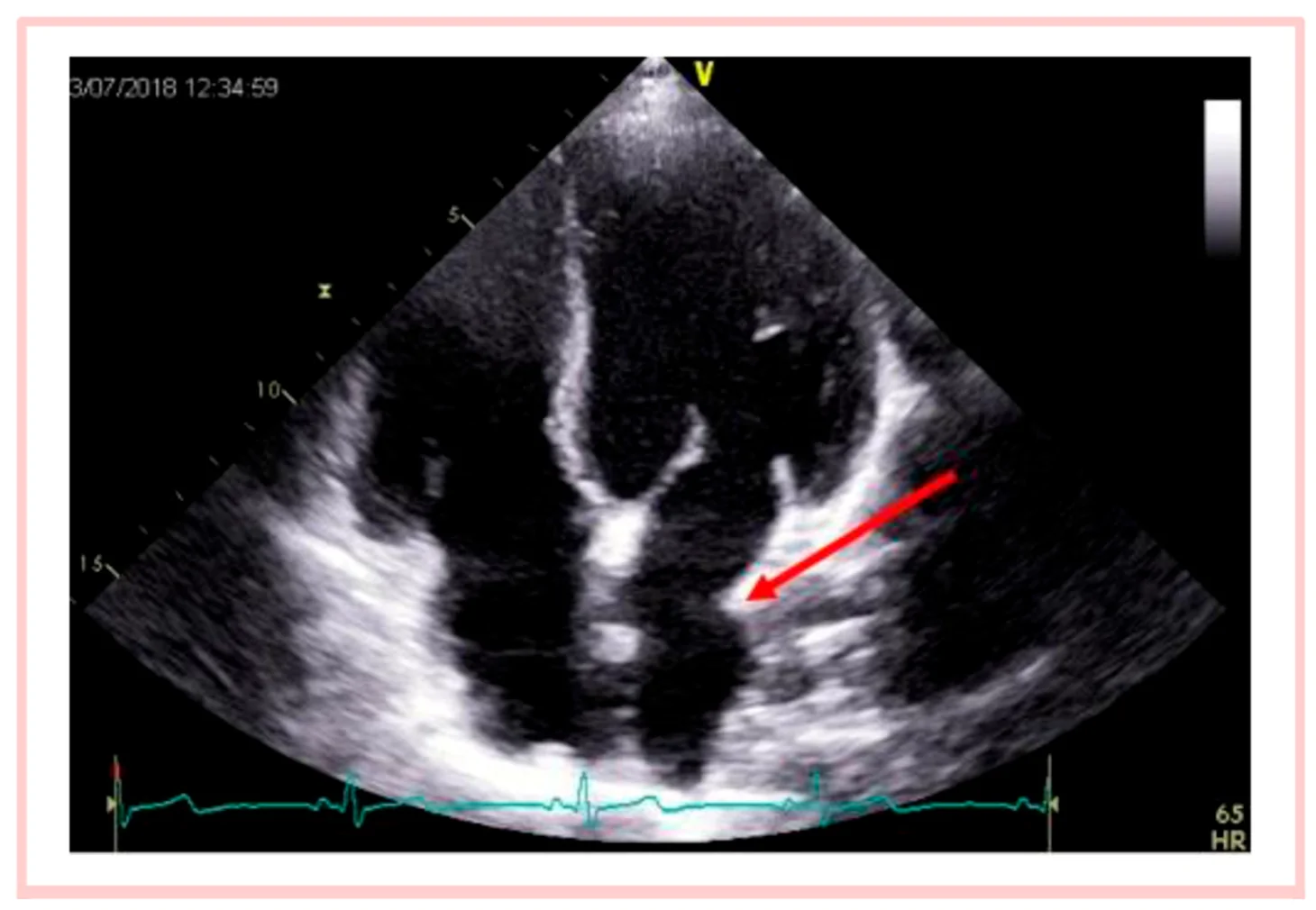
Apical four-chamber view, at trans thoracic echocardiography, after heart transplantation using a biatrial technique. Note the biatrial enlargement and the suture line in the left atrium that denotes the anastomosis between the donor and recipient atria (red arrow) [116].

The echocardiogram is another common tool that offers a 2D image of the heart. It is a relatively quick procedure that can be done in the outpatient setting without the use of radiation, though the quality of the images depends on operator skill and experience. Some echocardiographic findings of CAR include reduced left ventricular and/or right ventricular systolic function and increased left ventricular wall mass and thickness [Figure 3] [116,117,118]. Physiologic parameters, such as global longitudinal strain (GLS), have been employed for evaluation of ACR. For example, Masarone et al. found that GLS and RV longitudinal strain values were lower in rejection and correlated to a Grade 2R ACR [64,119]. A GLS <15% and RV longitudinal strain <17% were found to have high specificity, sensitivity, and negative predictive value but a poor positive predictive value of 25% and 42.9%, respectively. While Speckle tracking is another proposed physiologic test with a strong negative predictable value that could reduce the need for EMB, it does not have efficacy to detect rejection [120,121]. This is likely due in part to its use of tricuspid annular plane systolic excursion (TAPSE), which is known to be limited in assessing right ventricular function due to its inability to fully account for the ventricle’s complex asymmetry [122]. There is no echocardiographic finding sensitive or specific enough to replace EMB as the gold standard for diagnosing CAR, with a lack of data validating these anatomic and physiologic parameters as suitable markers for detecting rejection [120]. Nevertheless, echocardiography remains an important non-invasive tool for regular post-operative monitoring and HT prognosis.

#### 4.3.7. Positron Emission Tomography

Fludeoxyglucose-Positron Emission Tomography (FDG-PET) specializes in detecting areas of high metabolism and has been employed for infiltrative cardiomyopathy such as amyloidosis. An increase in FDG uptake is suggestive of an ongoing allograft rejection process [123,124]. The degree of uptake is classified into categories, where category 1 indicates no significant uptake and category 3 shows diffuse accumulation [123]. When compared to normal hearts, HTs undergoing a rejection event have higher rates of category 3 uptake and lower category 1 uptake compared to the control group. Apart from CAR, PET of myocardial blood flow (MBF) may have some utility in diagnosing CAV. It uses the ultrasound intravascular percent intimal volume (UIPIV) as a parameter, which is measured through a model that compares radiotracer activity versus time. A year post-HT, there was an increase in epicardial MBF that was postulated to be due to changes in the allograft microvasculature. This increase in MBF correlated with EMB findings of CAV [125]. Overall, PET-MBF can be a useful modality for early detection of CAV. However, more studies are needed to better elucidate the utility of PET in screening for CAV.

#### 4.3.8. Cardiac Magnetic Resonance Imaging

**Figure 4:**
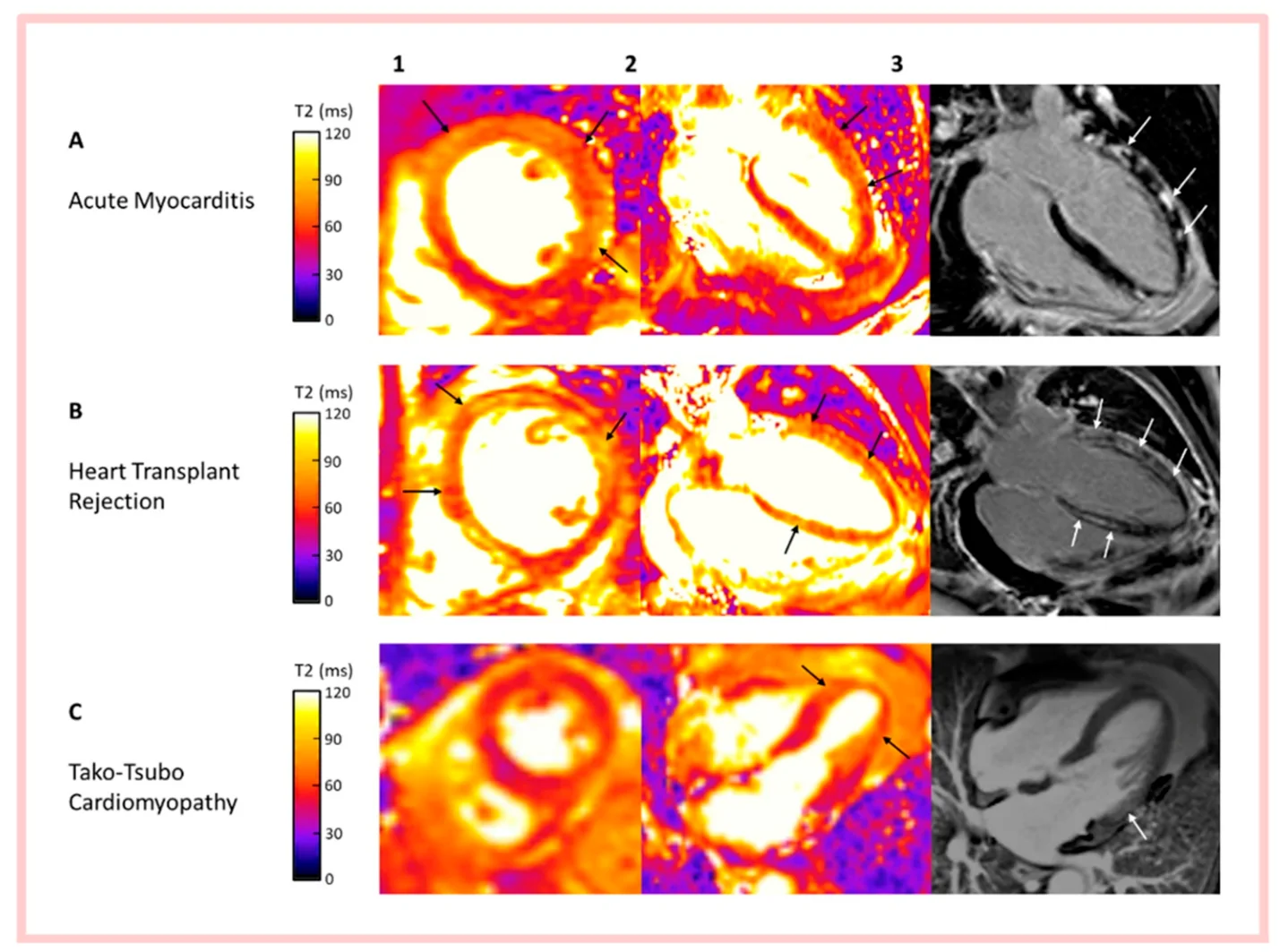
Cardiovascular Magnetic Resonance Imaging in patients with the presence of myocardial inflammation. Columns 1 and 2 depict T2 mapping, while column 3 shows late gadolinium enhancement. Column 1 shows a myocardial short-axis view with the left and right ventricle present. Columns 2 and 3 show a long-axis view, with arrows that point to certain areas of abnormal thickening of the myocardium, suggesting edema as a result of inflammation [127].

Cardiac magnetic resonance imaging (CMR) is the latest imaging modality that allows for better visualization of cardiac anatomy compared to echocardiograms without the variance of operator skill. The typical findings of CAR on CMR are prolonged T2 relaxation sequence, T1 hyper-enhancement with delayed enhancement, and elevated gadolinium uptake [Figure 4] [126,127]. These findings are suggestive of myocardial necrosis, edema, or fibrosis, which are suggestive of an ongoing CAR process [126]. A delayed T2 relaxation time is suggestive of a high water content in the myocardium, suggesting edema, which is typical of myocardial infarctions, myocarditis, and even acute rejection [128]. A meta-analysis found that a T2 relaxation time cutoff of 48.7–59 ms (mean 54.75) had a pooled sensitivity of 86.5%, specificity of 85.9%, positive likelihood ratio of 6.14, and negative likelihood ratio of 0.16 [129]. Furthermore, multiparameter CMR surveillance was found to be non-inferior to EMB surveillance [130]. The study also found a correlation with CMR findings and EMB for 2R rejections, and CMR had better detection of 1R rejection events. Furthermore, another study that compared CMR and EMB found that CMR had higher sensitivity and better negative predictive value at forecasting biopsy-proven rejection [131]. Late gadolinium enhancement (LGE) was also seen in HT patients with reduced ejection fraction but biopsy-negative rejection [93]. This pattern is associated with HF symptoms and hemodynamic instability. Ultimately, this finding is suggestive of inflammation, which could be a result of allograft rejection or a neurohormonal response, as concluded by the authors. Nevertheless, almost all patients in the study’s cohort received treatment and saw an improvement in their ejection fraction. While CMR has shown efficacy in detecting CAR and has a developing role as a surveillance tool, it has not been well studied for CAV. Small studies have been conducted looking at the diagnostic sensitivity for CAV using multiple parameters, including segmental T1, global T2, and extracellular volume (ECV). Among those with low-grade CAV, T2 and ECV were found to be the ideal parameters to evaluate [132]. T2 was also shown to be better compared to the other parameters at evaluating higher-grade CAV.

### 4.4. Short- and Long-Term Graft and Patient Management

#### 4.4.1. Hypertension

Hypertension is one comorbidity affecting those who undergo HT, affecting about 50–95% of recipients [133,134]. While many of these patients have pre-existing hypertension, post-transplantation renal toxicity secondary to calcineurin inhibitors is a contributing factor. While there are no randomized clinical trials looking into blood pressure regimens and goals for HT and their effects on graft function and longevity, it is postulated that blood pressure control is more beneficial in the HT recipient compared to the general population [89]. The common antihypertensives used for HT are calcium channel blockers (CCBs), angiotensin-converting enzyme inhibitors (ACEis), and angiotensin receptor blockers (ARBs) [89,134]. Out of these classes, ACEs and ARBs are preferred for their ability to inhibit maladaptive neurohormonal mechanisms contributing to hypertension.

#### 4.4.2. Hyperlipidemia

Dyslipidemia is another common disease that affects patients who received HT. It affects over 50% of patients, and if left untreated, it will cause atherosclerosis and CAV of the allograft’s coronary arteries, leading to graft dysfunction and failure [135]. Similar to other comorbid conditions, post-transplantation immunosuppressive agents such as calcineurin inhibitors and glucocorticoids are culprits in this disease. Much like in the general population, the medication of choice is statins. These medications have been shown to improve the short- and long-term outcomes of HT and reduce the incidence of CAV and rejection [136,137]. Other classes of lipid-lowering agents, such as ezetimibe and PCSK-9 inhibitors such as evolocumab, are being investigated to test their efficacy in this patient population. The EVOLVeD Trial analyzed evolocumab’s efficacy in reducing the progression of CAV in HT recipients compared to placebo. At the end of the 12-month study period, while evolocumab had a superior reduction in LDL compared to placebo, it had similar maximal intimal thickness as the placebo (mean 0.33 mm versus 0.30 mm) with a mean difference of 0.017 mm (*p* = 0.14) [137]. Despite the advantage of evolocumab in reducing LDL, it did not confer a practical benefit in halting the progression of CAV in HT.

#### 4.4.3. Diabetes Mellitus

Diabetes mellitus type 2 (DM2) is a common phenomenon after solid organ transplantation that appears in 4–25% of KT and 4–40% of HT patients [138]. Risk factors that contribute to the rise in DM2 include glucocorticoid use and insulin resistance driven by immunosuppressive agents such as tacrolimus and sirolimus [139]. Because DM2 carries a higher risk of decreased graft longevity, increased risk of infections, and worsened cardiovascular outcomes, strict glycemic control with insulin is recommended by the American Diabetes Association (ADA) and ISHLT in the immediate post-operative setting [140]. The ADA also recommends a post-HT goal HgbA1c of <7.0, though some have suggested a higher goal of between 7.5 and 8.0% due to post-operative hyperglycemia. It has also been suggested that Hgb_A1c_ may not accurately portray glycemic status due to concurrent anemia and renal impairment, requiring continuous glucose monitoring for more reliable surveillance [141]. Non-insulin medications such as metformin, thiazolidinediones, and sulfonylureas are generally discouraged due to their cardiovascular side effects and renal toxicity, especially in the case of metformin. Glucagon-like peptide 1 (GLP-1) agonists and sodium-glucose cotransporter 2 inhibitors (SGLT2is) are promising agents for long-term diabetes management. Randomized control trials studying their effects on HT recipients are scant, but small studies attest to their safety and cardiovascular benefit [142].

#### 4.4.4. Osteoporosis

Lastly, osteoporosis is another important condition to consider in the long-term management of HT. Its prevalence is unknown, but HT has the highest bone mineral loss out of all the different transplantations, with the most bone loss occurring in the first 3 months [143,144]. The most devastating complication of osteoporosis is pathologic fractures, which could lead to severe mobility and quality-of-life limitations. The pathogenesis of osteoporosis is multifactorial, with a decline in health condition and long-term use of glucocorticoids in particular being the main contributing factors [145]. The management of osteoporosis in HT is the same as it is in the general population. Resistance exercise and calcium and vitamin D supplementation form the backbone of the treatment strategy [146]. Bisphosphonates are especially important for reducing the progression of bone loss in those with severe osteoporosis or those who have had pathologic fractures.

## 5. Discussion

The finite number of available cardiac grafts to address an increasing cohort of ESHF patients who qualify for HT necessitates improvements in the allocation and treatment of candidates and recipients. Many of these advancements, such as policy changes that govern the timely allocation of donor hearts, enhancements to MCS devices that improve waitlist survival, and the monitoring and early detection of post-operative complications such as CAV, have reduced pre-operative and post-operative mortality. However, persistent challenges that remain include a recipient screening model with greater predictability for donor–recipient matching and post-operative outcomes, CAR monitoring as well as immunosuppressive optimization, and a shortage of available HT donors. To address these shortcomings, novel approaches like artificial intelligence (AI), xenoplantation and regenerative medicine are topics of current study that provide promising pathways to improve the evaluation and treatment of ESHF patients who are awaiting HT.

As previously discussed, there are many screening modalities to predict donor–recipient compatibility in HT candidates and post-operative outcomes. However, many of these models either underestimate or overestimate cardiac graft compatibility and mortality risk. Because of this, AI and machine learning (ML) are emerging tools that are currently under study to address this gap. In a retrospective study predicting 1-year HT post-operative mortality, multiple common ML models were shown to produce comparable outcomes to a traditional linear regression model [147]. Another recent study utilized ML to genetically screen HT candidates for known graft rejection markers to enable precise anti-rejection treatment [148]. This included predicting the appropriate dosing of immunosuppressive medications with the goal of tailoring treatment to the individual patient [149,150]. Despite these promising discoveries, AI still requires further development as it cannot autonomously develop predictive models and is prone to learning irrelevant patterns without guidance. Other barriers include the black box nature of AI, wherein the outcomes produced by ML cannot be entirely understood.

To address the deficit of available HT donor cardiac grafts, two novel approaches are xenotransplantation, or the transplantation of a non-human organ to a human recipient, and regenerative medicine. The recent groundbreaking work by Cooper et al. and the University of Maryland to transplant pig cardiac allografts into two separate patients, while short-lived (recipient survival was 60 days and 40 days, respectively), represents a possible avenue for future utilization [151,152]. Regarding the first candidate, the cardiac allograft remained viable after the hyperacute rejection period but later became inviable due to peri-operative complications (i.e., peri-operative aortic dissection and subsequent renal failure), the effects of humoral as well as cell-mediated rejection, and the reactivation of porcine cytomegalovirus and roseolovirus. Because of this, more research is needed to assess and optimize the targeting of the genetic biomarkers associated with rejection of the xenograft as well as the screening and selection of xenograft donors [153].

One new and innovative treatment for ESHF is regenerative medicine in the form of pluripotent stem cell therapy to replace non-functional cardiac tissue. Although there are relatively few clinical trials, one case report of a pluripotent stem cell patch that was implanted into the left ventricle (LV) of an ESHF patient showed symptomatic improvement, as well as an increased LV end-systolic volume index (LVESI) after 6 months; however, this decreased after 1 year [154]. Other similar studies showed post-procedural complications, most notably engraftment arrhythmia (EA) [155]. Ultimately, regenerative medicine remains a promising pathway to treat ESHF, but due to its recent emergence, it will require more expansive and robust study before it can be considered for widespread clinical application.

## 6. Limitations

One limitation of this literature review is the lack of rigor that is associated with systematic reviews, which employ frameworks such as Preferred Reporting Items for Systematic Reviews and Meta-Analysis (PRISMA). The literature was gathered using the online search engine PubMed via the University of Nevada, Las Vegas (UNLV) Libraries. The initial literature review took place from July to August 2025, and utilized direct key search terms including “heart failure”, “heart transplant”, and “post-transplant outcomes”. The results of this initial literature review (*n* = 149) were then appraised by the authors based on their relevance, with articles deemed as “high yield” due to their findings that directly contributed to the current and/or future practice of HT, “marginal yield” for studies with significant yet inconclusive findings that may influence further studies, and “low yield” if the research was considered superfluous and non-contributory with subsequent removal.

A framework for the literature review was then created based on the pre-operative, peri-operative, and post-operative periods of HT, as well as the references from the initial literature review. Further research was then added based on its relevance to the template and influence on the current and future practice of HT. These included studies were cited by organizations such as the ACC, AHA and ISHLT in their most recent guidelines for the management of HT candidates and recipients. Articles with pediatric data, with the exception those of Ingulli et al., Böhmer et al., and Das et al., were avoided to narrow the scope of the review to an adult population, with the aforementioned outliers cited due to their information on cellular rejection, dd-cfDNA, and imaging of CAR and CAV, respectively. The final draft and references were then reviewed by all of the authors, with independent as well as collective attempts to limit selection or confirmation biases as well as selective outcome reporting.

Despite these attempts, the lack of quantifiable or standardized inclusion and exclusion criteria maintained the risk for selection bias. The absence of a structured protocol reduces the clarity of the review, with the goal of featuring significant findings within the expansive literature emphasized over reproducibility. No formal or reliable assessment tools were employed to determine the quality of the incorporated research studies. The aforementioned studies are also limited in terms of small sample sizes and differing procedural aims, which restrict generalizability to a broader population.

Furthermore, the rapid evolution of the topics discussed in this review, including MCS device development, artificial intelligence (AI), xenotransplantation, and regenerative medicine, is a challenge which may hinder the recency of the research that was discussed. Because of this, several of the represented studies may not portray the most current advancements, with the possibility of new findings overruling the outcomes of this review. Despite these limitations, this literature review contributes an extensive and succinct summation of the current processes governing HT donor as well as recipient evaluation and management while suggesting future areas for further research.

## 7. Conclusions

Heart transplantation is a field that continues to evolve despite several enduring challenges, including donor scarcity, peri-operative risks inherent to this high-risk surgery, and long-term graft complications that can arise over time. Despite these challenges, it remains the gold standard therapy for patients with end-stage heart failure. We highlight the progress made in terms of candidate selection and organ allocation strategies, as well as advancements in mechanical circulatory support, peri-operative management, and patient surveillance post-organ transplant.

Certain risk stratification tools, prognostic scoring methods, and psychosocial assessments remain cornerstones in the candidate selection process. Newer innovations in mechanical circulatory support, such as LVADs and ECMO, improve candidate survival as bridges to transplantation. Additionally, enhancements in available immunosuppressive agents reduce overall cardiac allograft rejection rates, thereby shifting the field towards a less invasive approach to post-transplant monitoring. Modalities such as gene expression, donor-derived cell-free DNA, and advanced cardiac magnetic resonance imaging demonstrate promising accuracy in the early detection of graft dysfunction and rejection, reducing reliance on routine endomyocardial biopsy. Long-term complications like allograft vasculopathy, infection, and metabolic comorbidities still remain major causes of morbidity and mortality after transplantation, so future directions will include efforts to improve long-term patient outcomes by mitigating these complications.

Emerging technologies like xenotransplantation, regenerative medicine, and AI can help transform the field by increasing the potential donor pool, individualizing care, and improving graft longevity. Novel technologies alongside multidisciplinary team approaches with the implementation of advanced research will be critical to address existing limitations. We believe that heart transplantation will remain an effective therapy for patients with end-stage heart failure.

## Figures and Tables

**Table 1:** INTERMACS profiles of advanced heart failure.

INTERMACS Profile Description(s)	Timeframe for Intervention
Profile 1: Critical Cardiogenic Shock Critical cardiogenic shock despite escalating inotropic support—“Crash and burn”.	Definitive intervention needed within hours.
Profile 2: Progressive Decline Declining clinical status, either (1) despite inotropic therapy or (2) in patients who are unable to tolerate inotropic therapy—“Sliding on inotropes”.	Definitive intervention needed within hours.
Profile 2: Progressive decline	
Declining function despite inotropic support. Also describes declining status in patients unable to tolerate inotropic therapy. “Sliding on inotropes.”	Definitive intervention needed within a few days.
Profile 3: Stable but Inotrope-Dependent Hemodynamically stable while receiving either continuous inotropes, temporary circulatory support (e.g., ECMO), or both, but unable to stop support due to recurrent symptomatic hypotension or renal dysfunction—“Dependent stability.”	Definitive elective intervention over a period of weeks to several months.
Profile 4: Resting Symptoms/Recurrent Advanced Heart Failure Hemodynamically stable and near-normal volume status, but with daily symptoms including congestion at rest or during ADLs. Consider intensive management and surveillance strategies, which may reveal poor compliance that compromises the outcomes of any therapy. Note—some patients may alternate between 4 and 5—“Frequent flyer.”	Definitive elective intervention over a period of weeks to several months.
Profile 5: Exertion-Intolerant Comfortable at rest and with ADLs, but symptomatic with any physical activity. If underlying nutritional status and organ function are marginal, then the patient may be more at risk than if INTERMACS 4, and requires definitive intervention—“Housebound.”	Variable urgency—depends on maintenance of nutrition, organ function, and activity.
Profile 6: Exertion-Limited Comfortable at rest and with ADLs, along with minor activities (e.g., visiting friends), without evidence of fluid overload, but experiences fatigue within a few minutes of mild exertion. Requires careful measurement of peak oxygen consumption, in some cases with hemodynamic monitoring, to confirm the severity of cardiac impairment—“Walking wounded.”	Variable urgency—depends on maintenance of nutrition, organ function, and activity.
Profile 7: Advanced NYHA III Patients who are without current or recent episodes of unstable fluid imbalance, living comfortably with meaningful activity limited to mild physical exertion—“Placeholder”	Transplantation or circulatory support may not currently be indicated.
Profile 7: Advanced NYHA III	
Patients who are without current or recent episodes of unstable fluid balance, living comfortably with meaningful activity limited to mild physical exertion. “Placeholder”	Transplantation or circulatory support may not currently be indicated.

**Table 2:** Modifiers for INTERMACS profiles.

Modifiers for Profiles	Possible Profiles to Modify
TCS (temporary circulatory support) can modify only patients in hospital (other devices would be INTERMACS devices). Includes IABP, ECMO, TandemHeart, Levitronix, BVS 5000 or AB5000, Impella.	1, 2, 3 in hospital.
A—arrhythmia—can modify any profile. Recurrent ventricular tachyarrhythmias that have recently contributed substantially to clinical compromise. This includes frequent ICD shock or requirement for external defibrillator, usually more than twice weekly.	Any profile.
FF—frequent flyer—can modify only outpatients, designating a patient requiring frequent emergency visits or hospitalizations for diuretics, ultrafiltration, or temporary intravenous vasoactive therapy. At least two emergency visits/admissions in the past 3 months or three in the past 6 months	3 if at home, 4, 5, 6. A frequent flyer would rarely be profile 7.

**Table 3:** Changes in the 2018 UNOS heart transplant policy.

Adult Heart Allocation Criteria	
1999–2018	2018–Present
Status Categories	
Status 1A Admitted with TAH/IABP/ECMO LVAD with complications Continuous ventilation Continuous single or multiple inotropes requiring hemodynamic monitoring Able to discharge with LVAD for 30 days	Status 1 ECMO (up to 7 days) Unable to be discharged; surgically implanted non-endovascular BiV support device MCS with life-threatening ventricular arrhythmia
	Status 2 Unable to be discharged; surgically implanted non-endovascular LVAD (up to 14 days) IABP (up to 14 days) VT/VF, mechanical support not required MCS with device malfunction/mechanical failure TAH, BiVAD, RVAD, or VAD for single-ventricle patients Percutaneous endovascular MCS (up to 14 days)
	Status 3 May be discharged after LVAD implantation and re-evaluation within 30 days Multiple inotropes or single high-dose inotropes with continuous hemodynamic monitoring MCS with device infection, hemolysis, pump thrombosis, right heart failure, mucosal bleeding, or aortic insufficiency Temporary MCS after 14 days (7 days for ECMO) without reapproval
Status 1B All LVADs Continuous inotrope infusion	Status 4 Stable LVAD candidates not using 30-day discretionary period Inotropes without hemodynamic monitoring CHD, HCM/RCM, or amyloidosis IHD with intractable angina Re-transplantation
Status 2 All other listed candidates	Status 5 Combined organ transplants (awaiting at least 1 other organ at the same hospital)
	Status 6 All remaining active candidates
Geographic Changes	
Assigns donation service areas and concentric bands called “zones” to determine heart distribution Candidates are prioritized based on HLA sensitization Stratified by status and blood type	250 nautical mile fixed-distance circle from donor hospital HLA sensitization exception removed Stratified by status and blood type

BiV = biventricular; BiVAD = biventricular assist device; CHD = congenital heart disease; ECMO = extracorporeal membrane oxygenation; HCM = hypertrophic cardiomyopathy; HLA = human leukocyte antigen; IABP = intra-aortic balloon pump; IHD = ischemic heart disease; LVAD = left ventricular assist device; MCS = mechanical circulatory support; RCM = restrictive cardiomyopathy; RVAD = right ventricular assist device; TAH = total artificial heart; VAD = ventricular assist device; VF = ventricular fibrillation; VT = ventricular tachycardia; UNOS = United Network for Organ Sharing.

**Table 4:** SIPAT scoring sheet.

Psychosocial Domains		SIPAT Score
Category/Domain	Questions	
(A) Patient’s readiness level and illness management Score (0–24) ____	Knowledge and understanding of medical illness process (that caused specific organ failure)	0–4
	Knowledge and understanding of the process of transplantation	0–4
	Willingness/desire for treatment (transplant)	0–4
	History of treatment adherence/compliance (pertinent to medical issues)	0–8
	Lifestyle factors (including diet, exercise, fluid restrictions, and habits according to organ system)	0–4
(B) Social support system level of readiness Score (0–20) ____	Availability of social support system	0–8
	Functionality of social support system	0–8
	Appropriateness of physical living space and environment	0–4
(C) Psychological stability and psychopathology Score (0–37) _____	Presence of psychopathology (other than personality disorders and organic psychopathology)	0–8
	History of organic psychopathology or neurocognitive impairment (i.e., illness or medication-induced psychopathology)	0–5
	Influence of personality traits vs. disorder	0–4
	Effect of truthfulness vs. deceptive behavior	0–8
	Overall risk for psychopathology	0–4
(D) Lifestyle and effect of substance use Score (0–29) _____	Alcohol use, abuse, and dependence	0–8
	Alcohol abuse—risk for recidivism	0–4
	Illicit substance, abuse and dependence	0–8
	Illicit substance abuse—risk for recidivism	0–4
	Nicotine use, abuse, and dependence	0–5

**Table 5:** Heart transplant immunosuppressants and their side effects.

Class	Examples	Side Effects
Calcineurin Inhibitors	Cyclosporine Tacrolimus	Abdominal pain, nausea, vomiting, anorexia Electrolyte abnormalities Gingival hyperplasia Hypertension Infection and malignancy Nephrotoxicity
Antimetabolites	Azathioprine Mycophenolate mofetil	Abdominal pain, nausea, vomiting, anorexia Infection and malignancy
Glucocorticoids	Prednisone	Adrenal insufficiency Hyperglycemia Infection Metabolic syndrome Osteoporosis Psychosis
mTOR Inhibitors	Sirolimus Everolimus	Dermatitis Electrolyte abnormalities Hyperglycemia Mucositis

**Table 6:** ISHLT grades of acute cellular rejection.

Grade 0	No Rejection
Grade 1R	Interstitial and/or perivascular infiltrate with at least 1 area of myocyte damage
Grade 2R	2 or more areas of infiltration with myocardial damage
Grade 3R	Diffuse infiltration with or without edema, necrosis, vasculitis or hemorrhage

## Data Availability

No new data were created or analyzed in this study. Data sharing is not applicable to this article.
